# Trajectories of kidney function and risk of mortality

**DOI:** 10.1093/ije/dyad111

**Published:** 2023-08-30

**Authors:** Anna C van der Burgh, Sanaz Sedaghat, M Arfan Ikram, Ewout J Hoorn, Layal Chaker

**Affiliations:** Department of Internal Medicine, Erasmus Medical Center, University Medical Center Rotterdam, Rotterdam, The Netherlands; Department of Epidemiology, Erasmus Medical Center, University Medical Center Rotterdam, Rotterdam, The Netherlands; Department of Epidemiology and Community Health, School of Public Health, University of Minnesota, Minneapolis, Minnesota, USA; Department of Epidemiology, Erasmus Medical Center, University Medical Center Rotterdam, Rotterdam, The Netherlands; Department of Internal Medicine, Erasmus Medical Center, University Medical Center Rotterdam, Rotterdam, The Netherlands; Department of Internal Medicine, Erasmus Medical Center, University Medical Center Rotterdam, Rotterdam, The Netherlands; Department of Epidemiology, Erasmus Medical Center, University Medical Center Rotterdam, Rotterdam, The Netherlands

**Keywords:** Estimated glomerular filtration rate, trajectories, risk factors, latent class modelling, mortality

## Abstract

**Background:**

We aimed to identify patterns within the rate of kidney function decline, determinants of these patterns and their association with all-cause mortality risk in the general population.

**Methods:**

Participants aged ≥ 45 years with at least one assessment of creatinine-based estimated glomerular filtration rate (eGFR) taken between 1997 and 2018 were selected from a population-based cohort study. Analyses were performed using several distinct latent class trajectory modelling methods. Cumulative incidences were calculated with 45 years of age as the starting point.

**Results:**

In 12 062 participants (85 922 eGFR assessments, mean age 67.0 years, 58.7% women, median follow-up 9.6 years), four trajectories of eGFR change with age were identified: slow eGFR decline [rate of change in mL/min/1.73 m^2^ per year (RC), –0.9; 95% CI, –0.9 to –0.9; reference group], intermediate eGFR decline (RC, –2.5; 95% CI, –2.7 to –2.5) and fast eGFR decline (RC, –4.3; 95% CI, –4.4 to –4.1), and an increase/stable eGFR (RC, 0.3; 95% CI, 0.3 to 0.4). Women were more likely to have an increase/stable eGFR [odds ratio (OR), 1.94; 95% CI, 1.53 to 2.46] whereas men were more likely to have a fast eGFR decline (OR, 1.86; 95% CI, 1.33 to 2.60). Participants with diabetes, cardiovascular disease (CVD) or hypertension were more likely to have an intermediate or fast eGFR decline. All-cause mortality risks (cumulative incidence at age of 70 years) were 32.3% (95% CI, 21.4 to 47.9, slow eGFR decline), 6.7% (95% CI, 3.5 to 12.4, intermediate eGFR decline), 68.8% (95% CI, 44.4 to 87.8, fast eGFR decline) and 9.5% (95% CI, 5.5 to 15.7, increase/stable eGFR).

**Conclusion:**

Sex, hypertension, diabetes and CVD were identified as trajectory membership determinants. Having fast eGFR decline was associated with the highest risk of all-cause mortality, highlighting the need for extensive monitoring and prevention of kidney function decline in individuals at risk of having fast eGFR decline.

Key Messages

- This is the first study to investigate patterns within the rate of kidney function decline in the general population, together with the determinants and consequences of these patterns.
- We identified four trajectories of estimated glomerular filtration rate (eGFR) change with age, including a slow eGFR decline, an intermediate eGFR decline, a fast eGFR decline and an increase/stable eGFR trajectory.
- Sex, hypertension, diabetes and cardiovascular disease were identified as determinants of trajectory membership and the individual risk of chronic kidney disease and mortality could be determined by the pattern of eGFR change with age.

## Introduction

Chronic kidney disease (CKD) is generally considered to be an irreversible and progressive disease, characterized by worsening of kidney function expressed as a decrease in the estimated glomerular filtration rate (eGFR) and an increase in albuminuria over time.^1^ However, the rate of progression varies between individuals and depends on several characteristics such as age and sex.^2–4^ A less familiar concept is CKD regression. This term is used to describe an increase in kidney function over time in patients with CKD and has been described in several CKD populations.^5–8^ CKD regression could represent a true kidney function improvement resulting from, for example, risk factor treatment. However, CKD regression could also be related to kidney function-independent factors such as loss of muscle mass,^9^ which will especially play a role when CKD regression is diagnosed using eGFR based on serum creatinine. Hence, several patterns of kidney function change can occur in patients with CKD, from rapid kidney function decline to kidney function improvements, which has consequences for the individual risk of progression to kidney failure and mortality.^2^^,^^5^^,^^10^

A decrease in kidney function over time has been described not only in CKD patients, but in the general population as well. Recently, we reported an eGFR decline of 0.82 mL/min/1.73 m^2^ per year in middle-aged and elderly indivduals from the general population and several determinants of this decline were identified, including male sex and diabetes.^11^ However, it is still unknown whether distinct patterns of kidney function change exist in a general population as well. Currently, the burden of CKD, including its prevalence and mortality risk, is high and is expected to increase even further in the upcoming years despite extensive efforts to predict and prevent CKD.^12–14^ Therefore, there is a need for further improvements in CKD prediction and prevention. Identification of specific patterns of kidney function change in a general population and identifying determinants of these specific patterns could contribute to targeted prevention efforts. Furthermore, linking these patterns to mortality risk could provide insights into the clinical consequences of these specific patterns.

In the current study, we therefore aim to identify distinct patterns, also referred to as trajectories, within the rate of kidney function change over time within middle-aged and elderly individuals from a population-based cohort study. We also aim to explore determinants underlying these trajectories and to link the identified trajectories to the risk of all-cause mortality.

## Methods

A more detailed description of the Methods can be found in the Supplementary methods (available as Supplementary data at *IJE* online).

### Setting and study population

This study was conducted within the Rotterdam Study, an ongoing population-based cohort study from the Netherlands.^15^ Participants from the Rotterdam Study were eligible for the current study if they had a least one assessment of eGFR based on serum creatinine available within the Rotterdam Study or the Star-MDC database^11^ between baseline and end of follow-up. All participants were followed up from the day of baseline laboratory measurement to the date of death, loss to follow-up, the date of receiving dialysis or kidney transplantation, or the end of data collection on 24 May 2018, whichever came first. The Rotterdam Study has been approved by the Medical Ethics Committee of the Erasmus MC (registration number MEC 02.1015) and by the Dutch Ministry of Health, Welfare and Sport (Population Screening Act WBO, license number 1071272–159521-PG). Written informed consent was obtained from all study participants.

### Assessment of kidney function and covariates

Serum creatinine measurements within the Rotterdam Study and the Star-MDC database were performed using an enzymatic assay method. eGFR was calculated according to the Chronic Kidney Disease Epidemiology Collaboration (CKD-EPI) 2012 equation.^16^ Race was taken into account, although the vast majority of participants in the Rotterdam Study are of European descent (>97%). The baseline assessment of eGFR was defined as the first eGFR assessment available within the Rotterdam Study or the Star-MDC database after Rotterdam Study entry, from 1997 onwards. Information on the assessment of covariates can be found in the Supplementary methods (available as Supplementary data at *IJE* online). Urine albumin and creatinine were available at baseline for participants from the third Rotterdam Study cohort only, but for all participants at a follow-up visit. The urine albumin-to-creatinine ratio (ACR) was estimated by dividing urine albumin by urine creatinine (mg/g), where urine albumin and creatinine were determined by using a turbidimetric method and an enzymatic method, respectively, and measured using a Hitachi MODULAR P analyser (Roche/Hitachi Diagnostics, Mannheim, Germany).

### Assessment of mortality

Information on the vital status of all study participants was obtained from municipal health authorities in Rotterdam and through continuous digital linkage with records from general practitioners working in the study area. Information on cause of death was obtained from medical records of the general practitioners, hospitals and nursing homes. Two research physicians independently classified the cause of death according to the International Classification of Diseases, Tenth revision (ICD-10) and events were verified afterwards by a medical expert. Follow-up for mortality was complete until 24 May 2018.

### Statistical analysis

Our statistical analyses consisted of three consecutive steps, which included (i) determining the trajectories of eGFR change with age, (ii) characterizing subgroups following a specific trajectory and (iii) estimating the risk of mortality in the subgroups following a specific trajectory.

For the first step, we used latent class trajectory modelling to identify groups of participants with distinct trajectories of eGFR change with age. We calculated the individual posterior probabilities for each trajectory from the model and we assigned all individuals to the trajectory with the highest probability. The trajectories were labelled based on their specific pattern, to improve interpretability. Pre-defined stratification analyses by sex were performed. As a sensitivity analysis, we identified groups of participants with distinct trajectories of eGFR change with age, with eGFR calculated using the CKD-EPI 2021 equation.^17^ In a first post-hoc analysis, we adjusted the analyses for time-varying hypertension. In a second post-hoc analysis, we adjusted the analyses for time-varying use of renin–angiotensin–aldosterone system (RAAS) modifying agents.

For the second step, we added sex, prevalent hypertension, diabetes and CVD in a class-membership multinomial logistic model to the latent class trajectory model, to determine whether these covariates could explain trajectory membership. The slow eGFR decline trajectory was taken as the reference trajectory. Results were reported as odds ratios (ORs), representing the risk of having a specific eGFR trajectory, with their 95% CIs.

For the third step, we used a joint latent class model to describe the link between the distinct trajectories of eGFR change with age and all-cause mortality. The primary model was adjusted for age at baseline, sex and Rotterdam Study cohort (i.e. three cohorts) and the second model was additionally adjusted for the potential confounders body mass index, smoking, alcohol use, cholesterol and triglycerides. In a subsequent (third) model, we additionally adjusted for factors that could be both confounders as well as potential mediators including hypertension, prevalent CVD and diabetes. The results of the second and the third model were similar and we therefore only report the primary model and the most-adjusted model as our second model for all analyses. In the first post-hoc analysis, we adjusted the most-adjusted model for time-varying hypertension instead of baseline hypertension. In the second post-hoc analysis, we additionally adjusted the most-adjusted model for time-varying use of RAAS modifying agents. Class-specific cumulative incidences were calculated and plotted for the mean of covariates. Cumulative incidences were calculated with 45 years of age as a starting point and a Monte Carlo method was used to calculate 95% CIs. As a sensitivity analysis, we determined the link between the distinct trajectories of eGFR change with age and all-cause mortality by splitting follow-up time into two separate time periods. More details can be found in the Supplementary methods (available as Supplementary data at *IJE* online).

Multiple imputation was performed to impute missing values in the covariates. All analyses were performed using R statistical software [mice, JMBayes, lcmm and ggplot2 packages, R-project, R Foundation for Statistical Computing (2020), 3.6.3].

## Results

Overall, 12 062 participants were included in the study, with in total 85 922 repeated assessments of eGFR [with a median number of assessments of 5, interquartile range (IQR), 3; 10] (Supplementary Figure S1, available as Supplementary data at *IJE* online). The mean age of the population was 67.0 years and 58.7% were women (Table 1). The median duration of follow-up was 9.6 years (IQR, 7.0; 15.2).

**Table 1.** Baseline characteristics of the study population

Characteristic	**Total population (*N *=* *12** **062)**
Age, years (*n *=* *12 062)	67.0 ± 10.7
Sex, women (*n *=* *12 062)	7077 (58.7%)
BMI, kg/m^2^ (*n *=* *9829)	27.2 ± 4.2
Smoking, *n* (valid %) (*n *=* *10 052)	
Never	3293 (32.8%)
Past	4802 (47.8%)
Current	1957 (19.5%)
Alcohol, g/day (*n *=* *8272)	5.7 (0.5–14.3)
Serum cholesterol, mmol/L (*n *=* *9871)	5.7 ± 1.0
Serum triglyceride, mmol/L (*n *=* *9902)	1.5 ± 0.8
Systolic blood pressure, mmHg (*n *=* *9927)	140 ± 21
Diastolic blood pressure, mmHg (*n *=* *9927)	79 ± 12
Hypertension, *n* (valid %) (*n *=* *9902)	6250 (63.1%)
Diabetes, *n* (valid %) (*n *=* *10 337)	1246 (12.1%)
History of CVD, *n* (valid %) (*n *=* *10 286)	865 (8.4%)
Use of antihypertensive medication (*n *=* *9796)	3267 (33.4%)
Use of lipid-lowering medication (*n *=* *10 920)	1780 (16.3%)
Use of cardiac medication (*n *=* *10 920)	895 (8.2%)

Data are presented as number (%), mean ± SD or median (interquartile range). Values are given for non-imputed data. The *n* represents the number of participants without missing data. BMI, body mass index; CVD, cardiovascular disease; *n*, number.

### Characteristics of the eGFR trajectories

**Figure 1.**
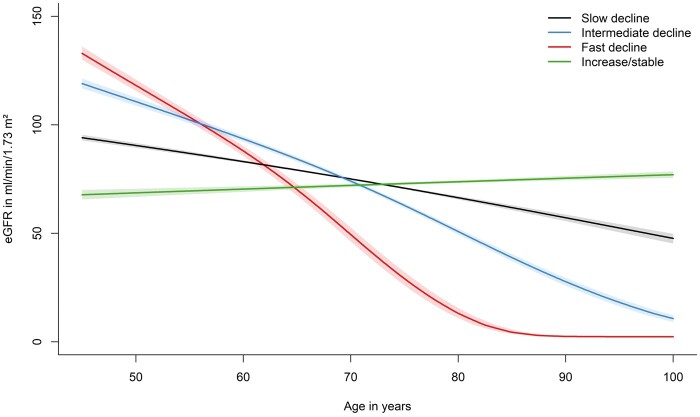
Trajectories of estimated glomerular filtration rate (eGFR) across age based on serum creatinine levels. eGFR: Estimated glomerular filtration rate

We identified four distinct trajectories of eGFR change with age (Figure 1 and Table 2). Three of the trajectories were characterized by a different rate of decline in eGFR, referred to as a slow (*n* = 8954, 74.2%), intermediate (*n* = 895, 7.4%) and fast decline (*n* = 222, 1.8%) in eGFR with age. The fourth trajectory was characterized by (slightly) increasing eGFR with age (*n* = 1991, 16.5%). The overall change in eGFR in mL/min/1.73 m^2^ per 1-year increase in age was –0.9 (95% CI, –0.9 to –0.9) for the slow decline, –2.5 (95% CI, –2.7 to –2.5) for the intermediate decline, –4.3 (95% CI, –4.4 to –4.1) for the fast decline and +0.3 (95% CI, 0.3 to 0.4) for the increase/stable trajectory. The results did not change substantially when calculating eGFR using the CKD-EPI 2021 equation or when adding hypertension or the use of RAAS modifying agents as time-varying covariates to the model (Supplementary Figures S2–S4, available as Supplementary data at *IJE* online). The median urine ACR at baseline was 3.5 (IQR, 2.2; 6.2) for the slow decline, 6.7 (IQR, 3.6; 17.0) for the intermediate decline, 5.5 (IQR, 3.3; 30.2) for the fast decline and 3.3 (IQR, 2.1; 6.3) for the increase/stable trajectory (Table 2). Similar summary statistics are shown for follow-up urine ACR (Table 2).

**Table 2.** Baseline characteristics of the study population separately for the determined trajectories (*N = *12 062)

Characteristic	Slow eGFR decline (*n = *8954)		Intermediate eGFR decline (*n = *895)		Fast eGFR decline (*n = *222)		Increase/stable eGFR (*n = *1991)	
	Summary statistic	*n*	Summary statistic	*n*	Summary statistic	*n*	Summary statistic	*n*
Age, years (*n = *12 062)	66.3 ± 11.1	8954	73.2 ± 9.1	895	65.4 ± 6.9	222	67.5 ± 9.3	1991
Sex, women (*n = *12 062)	5173 (57.8%)	8954	492 (55.0%)	895	94 (42.3%)	222	1318 (66.2%)	1991
BMI, kg/m^2^ (*n = *9829)	27.1 ± 4.2	7356	27.9 ± 4.0	669	28.9 ± 4.7	175	27.4 ± 4.2	1629
Smoking (*n = *10 052)		7500		705		185		1662
Never	2455 (32.7%)		211 (29.9%)		37 (20.0%)		590 (35.5%)	
Past	3540 (47.2%)		348 (49.4%)		92 (49.7%)		822 (49.5%)	
Current	1505 (20.1%)		146 (20.7%)		56 (30.3%)		250 (15.0%)	
Alcohol, g/day (*n = *8272)	6.3 (0.5–14.3)	6253	2.9 (0.1–14.3)	602	4.6 (0.1–15.0)	148	4.9 (0.5–15.0)	1269
eGFR, mL/min/1.73m^2^ (*n = *12 062)	79.1 ± 15.5	8954	69.1 ± 23.5	895	69.2 ± 28.7	222	73.1 ± 12.2	1991
Serum cholesterol, mmol/L (*n = *9871)	5.7 ± 1.0	7387	5.6 ± 1.1	675	5.6 ± 1.1	175	5.8 ± 1.0	1634
Serum triglyceride, mmol/L (*n = *9902)	1.5 ± 0.8	7412	1.7 ± 0.9	678	1.8 ± 1.0	174	1.5 ± 0.8	1638
Systolic blood pressure, mmHg (*n = *9927)	139 ± 21	7432	149 ± 23	683	147 ± 23	174	140 ± 21	1638
Diastolic blood pressure, mmHg (*n = *9927)	79 ± 11	7432	77 ± 12	683	80 ± 12	174	79 ± 11	1638
Hypertension (*n = *9730)	4466 (60.4%)	7939	584 (84.1%)	694	145 (80.6%)	180	1055 (64.5%)	1635
Diabetes, *n* (valid %) (*n = *10 337)	836 (10.8%)	7716	168 (22.9%)	735	68 (36.0%)	189	174 (10.3%)	1697
History of CVD (*n = *10 286)	600 (7.8%)	7676	109 (14.9%)	733	34 (18.1%)	188	122 (7.2%)	1689
Use of antihypertensive medication (*n = *9796)^a^	2217 (30.3%)	7316	352 (52.1%)	675	101 (57.1%)	177	597 (36.7%)	1628
Use of lipid-lowering medication (*n = *10 920)^a^	579 (7.1%)	8146	155 (20.1%)	772	31 (15.5%)	200	130 (7.2%)	1802
Use of cardiac medication (*n = *10 920)^a^	1282 (15.7%)	8146	128 (16.6%)	772	51 (25.5%)	200	319 (17.7%)	1802
Follow-up time, years (*n = *12 062)	8.9 (6.7–14.9)	8954	10.9 (6.7–15.3)	772	7.5 (5.0–10.5)	222	15.0 (10.2–16.5)	1991
Baseline urinary ACR, mg/g (*n = *3362)^b^	3.5 (2.2–6.2)	2940	6.7 (3.6–17.0)	68	5.5 (3.3–30.2)	51	3.3 (2.1–6.3)	303
Follow-up urinary ACR, mg/g (*n *=* *5945)^b^	3.6 (2.0–7.9)	4489	9.0 (4.1–31.2)	636	15.4 (2.7–62.9)	167	4.4 (31.2–10.1)	825
Number of repeated assessments from RS/total number of assessments (*n = *12 062)^c^	13 228/52 998 (25.0%)	8954	1108/10 112 (11.0%)	895	261/2080 (12.5%)	222	3309/20 732 (16.0%)	1991

Data are presented as number (%), mean ± SD, or median (interquartile range). Values are given for non-imputed data. The *n* represents the number of participants without missing data.

a The following World Health Organization Anatomical Therapeutic Chemical codes were used to define medication use: cardiac medication, C01; lipid-lowering medication, C10; and blood-pressure-lowering medication, consisting of antihypertensives, C02; diuretics, C03; beta blockers, C07; calcium-channel blockers, C08; and renin–angiotensin–aldosterone system modifying agents, C09.

b Urinary ACR was only available at baseline in participants from the third Rotterdam Study cohort. Data were available for participants from all three cohorts at a later visit during follow-up (collected between 2009 and 2015).

c Total number of repeated assessments = 85 922; total number of repeated assessments from the Rotterdam Study = 17 906; total number of repeated assessments from the Star-MDC database = 68 016.

ACR, albumin-to-creatinine ratio; BMI, body mass index; CVD, cardiovascular disease; eGFR, estimated glomerular filtration rate; *n*, number; RS, Rotterdam Study.

Similar trajectories were identified in males and females separately; however, in males, the fast eGFR decline trajectory started at a higher eGFR level and the increase/stable trajectory displayed a more stable pattern as compared with women (Supplementary Figure S5, available as Supplementary data at *IJE* online). The overall change in eGFR in mL/min/1.73 m^2^ per 1-year increase in age in males was –1.3 (95% CI, –1.3 to –1.3) for the slow decline, –2.8 (95% CI, –2.9 to –2.8) for the intermediate decline, –5.4 (95% CI, –5.7 to –5.1) for the fast decline and –0.0 (95% CI, –0.0 to 0.0) for the increase/stable trajectory. The overall change in eGFR in mL/min/1.73 m^2^ per 1-year increase in age in females was –0.8 (95% CI, –0.8 to –0.8) for the slow decline, –2.4 (95% CI, –2.4 to –2.3) for the intermediate decline, –4.1 (95% CI, –4.4 to –3.9) for the fast decline and 0.5 (95% CI, 0.4 to 0.5) for the increase/stable trajectory.

### Determinants of trajectory membership

By using a class-membership multinomial logistic model within the latent class trajectory model, we identified sex, hypertension, diabetes and prevalent CVD as determinants of the trajectory membership (Table 3). Women were more likely to have increasing/stable eGFR (OR, 1.94; 95% CI, 1.53 to 2.46) and less likely to have a fast eGFR decline (OR, 0.54; 95% CI, 0.38 to 0.75), both compared with having a slow eGFR decline. Conversely, men were more likely to have a fast eGFR decline (OR, 1.86; 95% CI, 1.33 to 2.60) and less likely to have increasing/stable eGFR (OR, 0.52; 95% CI, 0.41 to 0.65), both compared with having a slow eGFR decline.

**Table 3.** Association between several potential determinants explaining the trajectory membership of the four determined trajectories (slow decline, intermediate decline, fast decline and increase)

**Potential determinant** ^a^	Odds ratio (95% CI)
Sex, women	
Slow eGFR decline	Reference
Intermediate eGFR decline	0.96 (0.77–1.20)
Fast eGFR decline	0.54 (0.38–0.75)*
Increase/stable	1.94 (1.53–2.46)*
Sex, men	
Slow eGFR decline	Reference
Intermediate eGFR decline	1.03 (0.86–1.25)
Fast eGFR decline	1.86 (1.33–2.60)*
Increase/stable	0.52 (0.41–0.65)*
Hypertension	
Slow eGFR decline	Reference
Intermediate eGFR decline	2.55 (2.05–3.17)*
Fast eGFR decline	3.38 (2.14–5.35)*
Increase/stable	1.89 (1.51–2.35)*
Diabetes	
Slow eGFR decline	Reference
Intermediate eGFR decline	1.81 (1.45–2.26)*
Fast eGFR decline	4.65 (3.29–6.58)*
Increase/stable	1.27 (0.95–1.69)
Prevalent CVD	
Slow eGFR decline	Reference
Intermediate eGFR decline	1.53 (1.16–2.00)*
Fast eGFR decline	1.56 (0.99–2.48)
Increase/stable	1.31 (0.90–1.89)

a Women compared with men or vice versa; hypertension compared with no hypertension; diabetes compared with no diabetes; prevalent CVD compared with no prevalent CVD.

* *P *<* *0.05.

CVD, cardiovascular disease; eGFR, estimated glomerular filtration rate.

Participants with hypertension and participants with diabetes were more likely to have an intermediate eGFR decline (OR, 2.55; 95% CI, 2.05 to 3.17 for hypertension and OR, 1.81; 95% CI, 1.45 to 2.26 for diabetes) than to have a slow eGFR decline. Also, they were more likely to have a fast eGFR decline (OR, 3.38; 95% CI, 2.14 to 5.35 for hypertension and OR, 4.65; 95% CI, 3.29 to 6.58 for diabetes) than to have a slow eGFR decline. Participants with hypertension were also more likely to have increasing/stable eGFR (OR, 1.89; 95% CI, 1.51 to 2.35) as compared with having a slow a eGFR decline. Participants with prevalent CVD were more likely to have an intermediate eGFR decline (OR, 1.53; 95% CI, 1.16 to 2.00) than to have a slow eGFR decline.

### Risk of all-cause mortality by trajectory

**Figure 2.**
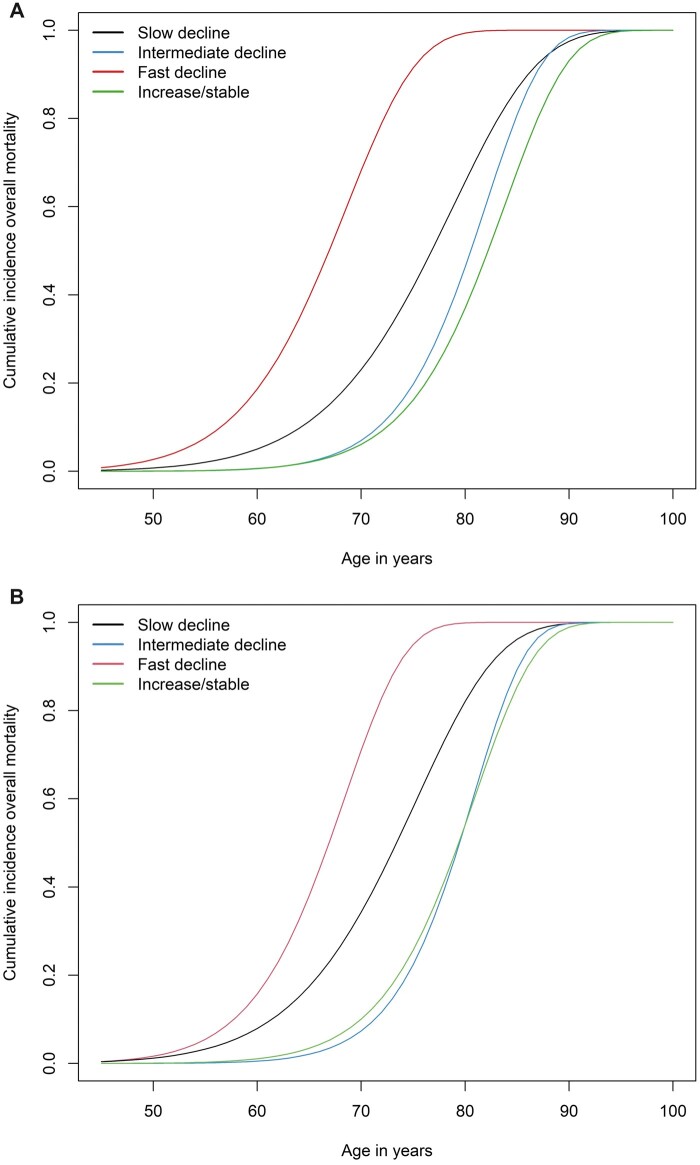
Four trajectories of estimated glomerular filtration rate (eGFR) based on serum creatinine levels and risk of all-cause mortality. (A) The survival part of the model is corrected for age at baseline, sex and Rotterdam Study cohort; (B) additionally adjusted for body mass index, smoking, alcohol use, cholesterol, triglycerides, hypertension, history of cardiovascular disease and diabetes. Cumulative incidences are plotted for the mean of covariates for continuous covariates and the reference category for categorical covariates

A total of 5277 deaths occurred during follow-up, which corresponds to an incidence rate of 41.7 per 1000 person-years. Overall, 3768 individuals with a slow eGFR decline, 637 individuals with an intermediate eGFR decline, 168 individuals with a fast eGFR decline and 704 individuals with increasing/stable eGFR died of any cause. Individuals with a fast eGFR decline had the highest cumulative incidence of all-cause mortality when compared with individuals in the other trajectories (68.8%; 95% CI, 44.4 to 87.8, up to the age of 70 years in the most-adjusted model; Figure 2B and Supplementary Table S1, available as Supplementary data at *IJE* online). The second-highest cumulative incidence of all-cause mortality was reported in individuals with a slow eGFR decline, with a cumulative incidence up to the age of 70 years of 32.3% (95% CI, 21.4 to 47.9) in the most-adjusted model (Supplementary Table S1, available as Supplementary data at *IJE* online).

When adjusted for age, sex and Rotterdam Study cohort, the cumulative incidence of all-cause mortality for individuals with an intermediate eGFR decline was higher compared with individuals with increasing/stable eGFR from the age of 65 years onwards (Figure 2A). In the most-adjusted model, cumulative incidences of both groups became similar, although individuals with increasing/stable eGFR up to the age of 70 years had slightly higher cumulative incidence (9.5%; 95% CI, 5.5 to 15.7 for the eGFR increase/stable trajectory vs 6.7%; 95% CI, 3.5 to 12.4 for the intermediate decline eGFR trajectory). All results did not change substantially when adding hypertension or RAAS modifying agents use as time-varying covariates to the model (Supplementary Table S2, available as Supplementary data at *IJE* online).

### Sensitivity analysis

We identified three trajectories of eGFR change with age during the first 10 years of follow-up (Supplementary Figure S6, available as Supplementary data at *IJE* online). The overall change in eGFR in mL/min/1.73 m^2^ per 1-year increase in age was –0.3 (95% CI, –0.3 to –0.2) for the increase/stable, –1.1 (95% CI, –1.1 to –1.0) for the slow decline and –2.4 (95% CI, –2.5 to –2.3) for the intermediate/fast decline trajectory. Individuals with a fast eGFR decline had a higher all-cause mortality risk compared with individuals with a slow eGFR decline (HR 1.71; 95% CI, 1.40 to 2.10, Supplementary Table S3, available as Supplementary data at *IJE* online).

## Discussion

In this prospective population-based cohort study of 12 062 middle-aged and elderly individuals from the general population, we identified four distinct trajectories of eGFR change with age using latent class analysis, characterized by a slow, intermediate and fast eGFR decline and by increase/stable eGFR. We identified sex, hypertension, diabetes and prevalent CVD as determinants of trajectory membership. All-cause mortality was highest in individuals with a fast eGFR decline. Surprisingly, individuals with a slow eGFR decline had the second-highest all-cause mortality rate.

A rapid eGFR decline in the general population has been previously described as a decline of 3 mL/min/1.73 m^2^ per year,^18–20^ although we showed that we could further divide this into an intermediate (decline of 2.6 mL/min/1.73 m^2^ per year) and fast eGFR decline (decline of 4.4 mL/min/1.73 m^2^ per year), for which the mortality risks also differ. Various previous studies also focused on unravelling the different patterns of eGFR change over time, but showed conflicting results and were mainly performed in individuals with CKD, patients with diabetes or in kidney transplant recipients.^21–29^ One previous study of trajectories was conducted in elderly individuals aged between 65 and 90 years attending annual geriatric health checkups in Japan.^30^ Although they identified three trajectories, these only differed in the absolute eGFR value with age, whereas no clear difference in the rate of decline by age was identified, which is contradictory to our findings and previous other literature.

Recently, the concept of CKD regression has been introduced^5–8^ and our latent class analysis also identified a trajectory with a slight increase in eGFR over time, similar to the concept of CKD regression. The underlying pathophysiology and meaning are unclear, but it could be hypothesized that this increase in eGFR with age is related to a more progressive decline in skeletal muscle mass, also referred to as sarcopenia, than nephron loss with age.^31^ Serum creatinine largely depends on muscle mass^32^ and therefore decreased muscle mass will cause a decrease in serum creatinine and an increase in eGFR based on serum creatinine. This potential confounding effect of muscle mass can be bypassed when using serum cystatin C to determine eGFR, as serum cystatin C is less affected by changes in muscle mass.^33^^,^^34^ However, it should be noted that participants with an increase in eGFR were more often women and had a relatively lower risk of all-cause mortality. Previous studies have shown that the age-related rate of muscle loss is higher in men as compared with women^35^ and that individuals with sarcopenia have an increased risk of mortality.^36^ This suggests that solely the presence of sarcopenia and muscle mass loss is unlikely to fully explain the increase in eGFR with age and other mechanisms might play a role as well. Mechanisms underlying CKD regression other than loss of muscle mass might include changes in lifestyle factors, renoprotective treatment effects or spontaneous recovery of mild CKD.^9^^,^^37^ Whether these and potential other mechanisms play a role in the eGFR increase in the general population as well should be investigated further.

Several previous studies have reported an increased mortality risk with decreased kidney function.^38–40^ Moreover, there even seems to be a graded association between the rate of eGFR decline and mortality risk.^2^^,^^10^^,^^18^^,^^41^ In the current study, however, having a slow eGFR decline, and not having an intermediate eGFR decline, was associated with the second-highest risk of all-cause mortality. Yet, individuals with an intermediate eGFR decline had a higher prevalence of hypertension, diabetes and CVD compared with individuals with a slow eGFR decline. At the same time, these individuals were also more often treated with antihypertensive and lipid-lowering medication, which could have lowered the risk of mortality in this group of individuals.^42–46^ Treatment with these medications could have modified the association between eGFR decline and mortality, explaining why having an intermediate eGFR decline was associated with a lower mortality risk compared with having a slow eGFR decline. Additionally, we speculate that individuals with an intermediate decline might be detected sooner and, because their decline is still in the intermediate range, therapeutic interventions might be in time to improve prognosis. A slow eGFR decline may not lead to treatment recommendations because this pattern of decline is considered to be caused by healthy ageing. Whether differential treatment benefits underlie the identified differences in the association with mortality risk between slow and intermediate eGFR decline could be of interest for further research. Our sensitivity analysis in which we split follow-up time showed similar trajectories. However, due to the decrease in the number of events using this method, we were not able to distinguish between fast and intermediate decline. Furthermore, using this method could have introduced immortal time bias.

In the current study, we identified sex as one of the determinants of trajectory membership. We showed that women were more likely to have increasing/stable eGFR and less likely to have a fast eGFR decline, whereas opposite results were shown in men. It has been previously described that women have a higher prevalence of CKD, whereas men show a more rapid CKD progression.^47^^,^^48^ A similar pattern was previously identified in the general population, in which men were more likely to have a faster eGFR decline with age.^11^ We also report that women were more likely to have increasing/stable eGFR, or eGFR regression, which might imply a decreased risk of developing CKD in women. These findings were supported by the trajectory analyses performed in males and females separately, as we revealed that the fast decline trajectory started at a higher eGFR level and declined faster with age in males compared with females. In addition, we identified an increase/stable trajectory in females, but a stable trajectory in males. These findings could serve as a possible first step in the application of sex-specific prevention and treatment strategies for eGFR decline.

We also identified hypertension, diabetes and prevalent CVD as determinants of trajectory membership, which are well-established risk factors for kidney function decline and CKD.^11^^,^^49–52^ As expected, participants with these comorbidities were more likely to have an intermediate or fast decline in eGFR with age. However, participants with hypertension were also 1.9 times more likely to have increasing/stable eGFR compared with having a slow decline in eGFR. A potential explanation for this unexpected finding is the presence of glomerular hyperfiltration. The association between diabetes and hyperfiltration is well established.^45^ Although an association between hypertension and hyperfiltration has been suggested as well, this is still largely unknown.^53–55^ The presence of hyperfiltration might also explain why having increasing/stable eGFR is associated with higher all-cause mortality risks as compared with having an intermediate eGFR decline. However, proteinuria is an important characteristic of hyperfiltration and, in the current study, the urine ACR was the lowest in participants with an increase/stable eGFR and in participants with a slow eGFR decline. Therefore, other explanations of our findings should also be considered, such as a confounding role of muscle mass, as changes in muscle mass affect serum creatinine^32^ and might also affect blood pressure,^56–58^ or the presence of renoprotective treatment effects.^59^^,^^60^ Therefore, in clinical practice, glomerular hyperfiltration and other potential non-eGFR determinants should be taken into account when evaluating eGFR in individuals with hypertension.

The strengths of our study include the large sample size from the general population, the high number of repeated assessments of eGFR over time and the prospective design with a long follow-up period, which enabled us to identify different eGFR trajectories using a latent class trajectory analysis. This allowed us to identify four distinct and clinically relevant trajectories of eGFR change with age. Furthermore, the extensive information on covariates enabled us to control for several potential confounders, although unmeasured and residual confounding cannot be fully excluded in an observational study. A main limitation of our study is that our population mainly includes individuals from European descent aged 45 years or older, which limits the generalizability of our findings to other populations. A second limitation of our study is the unavailability of glomerular filtration rate (GFR) measurements based on the clearance of exogenous filtration markers, which is the ‘golden standard’ for assessing GFR. However, performing these measurements is not feasible in a large population study nor standard in clinical care. Therefore, information on eGFR trajectories instead of on GFR trajectories will still provide the most valuable information for clinical practice. A third limitation is that information on acute kidney injury is not available, although inspection of participants’ individual eGFR declines did not reveal any sharp declines in eGFR pointing towards episodes of acute kidney injury. A fourth limitation is that we could not draw any final conclusions about the presence or absence of hyperfiltration due to the unavailability of repeated ACR measurements.

In conclusion, our results indicate the potential presence of an individual risk of CKD and mortality. This individual risk could be determined by the pattern of eGFR change with age. This knowledge, together with the identification of determinants of trajectory membership, may contribute to improving the prediction and even the prevention of CKD and early mortality. Having a fast eGFR decline was associated with the highest risk of all-cause mortality, implicating the need for extensive monitoring of individuals at higher risk of having a fast eGFR decline. Future studies are however needed to investigate the causality of the identified associations between the distinct trajectories and all-cause mortality risks. Furthermore, as proteinuria is an important aspect of CKD next to eGFR decline, investigating trajectories of proteinuria with age in future research is needed.

## Ethics approval

The Rotterdam Study complies with the Declaration of Helsinki and has been approved by the Medical Ethics Committee of the Erasmus MC (registration number MEC 02.1015) and by the Dutch Ministry of Health, Welfare and Sport (Population Screening Act WBO, license number 1071272–159521-PG).

## Supplementary Material

dyad111_Supplementary_Data

## Data Availability

The data sets analysed during the current study are not publicly available due to legal and ethical restraints. Data are available from the corresponding author on reasonable request.
