# Supplementary material for: Trajectories of kidney function and risk of mortality
Source: Int J Epidemiol. 2023 Aug 30;52(6):1959–67. doi: 10.1093/ije/dyad111 (PMC10749765; doi:10.1093/ije/dyad111)
Supplement: dyad111_Supplementary_Data [file dyad111_supplementary_data.docx]

**Supplementary Materials**

**Supplementary Methods**

**Supplementary Table S1.** Cumulative incidences of all-cause mortality for trajectories of eGFR

**Supplementary Table S2.** Cumulative incidences of all-cause mortality for trajectories of eGFR, adjusted for time-varying hypertension and time-varying use of RAAS modifying agents

**Supplementary Table S3.** All-cause mortality risk for trajectories of eGFR

**Supplementary Figure S1.** Flowchart of the study population

**Supplementary Figure S2.** Trajectories of eGFR across age, with eGFR calculated with the CKD-EPI 2021 equation based on serum creatinine levels

**Supplementary Figure S3.** Trajectories of eGFR across age based on serum creatinine levels, taking hypertension as time-varying covariate into account.

**Supplementary Figure S4.** Trajectories of eGFR across age based on serum creatinine levels, taking the use of RAAS modifying agents as time-varying covariate into account.

**Supplementary Figure S5.** Trajectories of eGFR across age based on serum creatinine levels, separately for males and females.

**Supplementary Figure S6.** Ten-year trajectories of eGFR across age

**Supplementary Figure S7.** Longitudinal evolution of eGFR

**Supplementary Methods**

**Setting and study population**

This study was conducted within the Rotterdam Study, an ongoing population-based cohort study from the Netherlands (1). The study started in 1990 and originally included 7,983 participants from Ommoord aged > 55 years, a suburb of Rotterdam. This original cohort was extended with a second (aged >55 years ) and a third cohort (aged > 45years) in 2000 and 2006, respectively, resulting in a total study population of 14 926 by the end of 2008. The average response rate was 72%. Participants were examined at baseline and follow-up examinations are conducted every 3-6 years. Additionally, the total population is continuously monitored for relevant outcomes. Participants from the Rotterdam Study were eligible for the current study if they had a least one assessment of eGFR based on serum creatinine available within the Rotterdam Study or the Star-MDC database between baseline and end of follow-up. The Star-MDC database is a database from a center for medical diagnostics for outpatients in the city of Rotterdam. We have previously described that exploration of the general practitioner data reveals high similarities with the Rotterdam Study data and because of this, we do not expect that bias is introduced when using the general practitioner data. For example, participants with a high number of assessments were not the participants with worse kidney function and findings were similar when using data from the Rotterdam Study and when using data from the Star-MDC database (Supplementary Figure S7). Baseline kidney function was similar in participants with >5 repeated assessments versus participants with ≤ 5 assessments (77.4 ± 17.3 versus 76.9 ± 15.5). Further details have been published elsewhere (2). Baseline was defined as the third visit of cohort 1 (1997-1999), the first visit of cohort 2 (2000-2001), and the first visit of cohort 3 (2006-2008). All participants were followed up from the day of baseline laboratory measurement to the date of death, loss-to-follow-up, the data of receiving dialysis or kidney transplantation, or to the end of data collection on May 24^th^, 2018, whichever came first. The Rotterdam Study has been approved by the Medical Ethics Committee of the Erasmus MC (registration number MEC 02.1015) and by the Dutch Ministry of Health, Welfare and Sport (Population Screening Act WBO, license number 1071272-159521-PG). The Rotterdam Study Personal Registration Data collection is filed with the Erasmus MC Data Protection Officer under registration number EMC1712001. The Rotterdam Study has been entered into the Netherlands National Trial Register (NTR; www.trialregister.nl) and into the WHO International Clinical Trials Registry Platform (ICTRP; www.who.int/ictrp/network/primary/en/) under shared catalogue number NTR6831. Written informed consent was obtained from all study participants.

**Assessment of kidney function**

Serum creatinine measurements within the Rotterdam Study and the Star-MDC database were performed using an enzymatic assay method (Roche/Hitachi analyzers, Roche/Hitachi Diagnostics, Mannheim, Germany). Serum collection, storage, and measurements of creatinine were performed in a similar manner within the Rotterdam Study and the Star-MDC database. Creatinine values were standardized to isotope-dilution mass spectrometry–traceable (ID-MS) measurements. In addition, creatinine was calibrated by aligning the mean serum creatinine values from our cohort with those of the Third National Health and Nutrition Examination Survey (NHANES III) in age categories of <50, 50-59, 60-69, ≥70, separately for men and women. For calibration, a CFAS calibrator (Calibrator for automated systems) was used. There was no lot-to-lot variation. eGFR was calculated according to the Chronic Kidney Disease Epidemiology Collaboration (CKD-EPI) equation (3). Race was taken into account, however the vast majority of participants in the Rotterdam Study are from European descent (>97%). The baseline assessment of eGFR was defined as the first eGFR assessment available within the Rotterdam Study or the Star-MDC database after Rotterdam Study entry, from 1997 onwards. Urine albumin and creatinine were available at baseline for participants from the third Rotterdam Study cohort only, but for all participants at a follow-up visit. The urine albumin-to-creatinine ratio (ACR) was estimated by dividing urine albumin by urine creatinine (mg/g), where urine albumin and creatinine were determined by a turbidimetric method and an enzymatic method, respectively, and measured by a Hitachi MODULAR P analyzer (Roche/Hitachi Diagnostics, Mannheim, Germany).

**Assessment of mortality**

Information on vital status of all study participants was obtained from municipal health authorities in Rotterdam and through continuous digital linkage with records from general practitioners working in the study area. Information on cause of death was obtained from medical records of the general practitioners, hospitals, and nursing homes. Two research physicians independently classified the cause of death according to International Classification of Diseases, Tenth revision (ICD-10) and events were verified afterwards by a medical expert. Follow-up for mortality was complete until May 24^th^ 2018.

**Covariates and determinants**

Information on demographics, alcohol intake, tobacco smoking, and medication use was obtained through home questionnaires. The average daily consumption of alcohol was reported by the participants and converted to grams per day. Tobacco smoking was categorized in never, past, and current smoking. To define the use of cardiac medication, lipid-lowering medication, and blood-pressure lowering medication, the following WHO's Anatomical Therapeutic Chemical (ATC) codes were used: cardiac medication (C01), lipid-lowering medication (C10), and blood-pressure lowering medication, including antihypertensives (C02), diuretics (C03), beta blockers (C07), calcium channel blockers (C08), and renin-angiotensin-aldosterone system (RAAS) modifying agents (C09)). Height and weight were measured at the research center. Body mass index (BMI) was calculated by dividing weight in kilograms by height in meters squared. Serum cholesterol and triglycerides were determined using standard laboratory techniques. Systolic and diastolic blood pressure were measured on the right arm using a random-zero sphygmomanometer and the mean of two consecutive measurements was taken as the final measurement. Hypertension was defined as elevated systolic or diastolic blood pressure (exceeding 140/90 mmHg) or the use of antihypertensive medication prescribed for the indication of hypertension. Diabetes mellitus diagnosis was based on a fasting serum glucose level ≥ 7.0 mmol/l, a non-fasting serum glucose level ≥ 11.1 mmol/l (when fasting samples were absent), the use of blood glucose lowering medication or a previous diagnosis of the disease. Prevalent cardiovascular disease (CVD) was defined as a history of myocardial infarction, stroke, and coronary or other arterial revascularization (4, 5), and assessed during the baseline home interviews and verified by clinical data from the medical records (4).

**Statistical analysis**

Our statistical analyses consisted of three consecutive steps, which included 1) determining the trajectories of eGFR change with age, 2) characterizing subgroups following a specific trajectory, and 3) estimating the risk of mortality in the subgroups following a specific trajectory.

For the first step, we used latent class trajectory modeling to identify groups of participants with distinct trajectories of eGFR change with age. The optimal number of trajectories was determined by the lowest Bayesian Information Criterion (BIC), while maintaining the posterior probabilities of each class above 0.70 (6). The link function was chosen using similar criteria and I-splines with 3 nodes located at the tertiles of the eGFR distribution provided the best fit. Age was used as the time variable. Non-linearity was tested by adding natural cubic splines to the model. The best fit was determined to be linear. Only random intercepts were included, as adding random slopes did not improve the model fit. The analysis was repeated for different sets of random initial values, to ensure convergence to the global maximum of the model (7-9). We calculated the individual posterior probabilities for each trajectory from the final model and we assigned all individuals to the trajectory with the highest probability. The trajectories were labelled based on their specific pattern, to improve interpretability. We performed pre-defined stratification analyses by sex. As a sensitivity analysis, we identified groups of participants with distinct trajectories of eGFR change with age, with eGFR calculated using the CKD-EPI 2021 equation (10). In a first post-hoc analysis, we adjusted the analyses for time-varying hypertension. In a second post-hoc analysis, we adjusted the analyses for time-varying use of RAAS modifying agents.

For the second step, we added sex, hypertension, diabetes, and prevalent CVD in a class-membership multinomial logistic model to the latent class trajectory model, to determine whether these covariates could explain trajectory membership. All covariates were determined at baseline. Two models were created, one with men and one with women as the reference category of sex, in order to investigate sex-specific differences in more detail. Generally, a decline in glomerular filtration rate and eGFR of around 0.8 ml/min/1.73 m^2^ per year is considered as an average decline of eGFR with healthy aging (2, 11-14). Therefore, for these analyses, the trajectory that approximates the decline in eGFR observed with healthy aging was taken as reference and is labelled slow eGFR decline trajectory. Results were reported as odds ratios (ORs), representing the risk of having a specific eGFR trajectory, with their 95% confidence intervals (CIs).

For the third step, we used a joint latent class model to describe the link between the distinct trajectories of eGFR change with age and all-cause mortality. This model assumes that a population is heterogeneous and consists of several sub-populations which can be characterized by distinctive patterns of the evolution of a marker of interest, such as eGFR, and which could have different risks of the event, such as mortality (15). Age was used as the time variable and potential left truncation was taken into account in the analyses. The primary model was adjusted for age at baseline, sex, and Rotterdam Study cohort and the second model was additionally adjusted for the potential confounders BMI, smoking, alcohol use, cholesterol, and triglycerides. In a subsequent (third)model, we additionally adjusted for factors that could be both confounders as well as potential mediators including hypertension, prevalent CVD, and diabetes. For selection of the final model, the same criteria as reported in the first step were used. The results of the second and third model were similar and we therefore only report the primary model and the most adjusted model as our second model for all analyses. In the first post-hoc analysis, we adjusted the most adjusted model for time-varying hypertension instead of baseline hypertension. In the second post-hoc analysis, we additionally adjusted the most adjusted model for time-varying use of RAAS modifying agents. Class-specific cumulative incidence were calculated and plotted for the mean of covariates. Cumulative incidences were calculated with 45 years of age as a starting point and a Monte Carlo method was used to calculate 95% CIs. Cumulative incidences up to the age of 70 years is reported. Cumulative incidences up to other ages are included in the tables. As a sensitivity analysis, we determined the link between the distinct trajectories of eGFR change with age and all-cause mortality by splitting follow-up time into two separate time periods. We used latent class trajectory modeling to identify trajectories of eGFR change with age using the first ten-years of follow-up. Subsequently, Cox-proportional hazards models were applied to calculate the HRs for all-cause mortality by assigned eGFR trajectory, where the start of follow-up was defined as the date of ending follow-up for trajectory definition. The proportional-hazards assumption was checked using the Schoenfeld test and by assessing the Schoenfeld plot.

For all analyses, covariates were considered to be baseline covariates when their information was collected within six months from the start date, otherwise they were classified as missing (14% in total) and imputed taking the nearest set of covariates available within the Rotterdam Study taken into account. Multiple imputation was performed to impute missing values in the covariates. All analyses were performed using R statistical software (mice, JMBayes, lcmm and ggplot2 packages, R-project, R Foundation for Statistical Computing (2020), 3.6.3).

References

1. Ikram MA, Brusselle G, Ghanbari M, Goedegebure A, Ikram MK, Kavousi M, et al. Objectives, design and main findings until 2020 from the Rotterdam Study. Eur J Epidemiol. 2020.

2. van der Burgh AC, Rizopoulos D, Ikram MA, Hoorn EJ, Chaker L. Determinants of the Evolution of Kidney Function With Age. Kidney International Reports. 2021;6(12):3054-63.

3. Inker LA, Schmid CH, Tighiouart H, Eckfeldt JH, Feldman HI, Greene T, et al. Estimating glomerular filtration rate from serum creatinine and cystatin C. N Engl J Med. 2012;367(1):20-9.

4. Leening MJ, Kavousi M, Heeringa J, van Rooij FJ, Verkroost-van Heemst J, Deckers JW, et al. Methods of data collection and definitions of cardiac outcomes in the Rotterdam Study. Eur J Epidemiol. 2012;27(3):173-85.

5. Wieberdink RG, Ikram MA, Hofman A, Koudstaal PJ, Breteler MM. Trends in stroke incidence rates and stroke risk factors in Rotterdam, the Netherlands from 1990 to 2008. Eur J Epidemiol. 2012;27(4):287-95.

6. Lennon H, Kelly S, Sperrin M, Buchan I, Cross AJ, Leitzmann M, et al. Framework to construct and interpret latent class trajectory modelling. BMJ Open. 2018;8(7):e020683.

7. Biernacki C, Celeux G, Govaert G. Choosing starting values for the EM algorithm for getting the highest likelihood in multivariate Gaussian mixture models. Computational Statistics & Data Analysis. 2003;41(3):561-75.

8. Proust-Lima C, Sene M, Taylor JM, Jacqmin-Gadda H. Joint latent class models for longitudinal and time-to-event data: a review. Stat Methods Med Res. 2014;23(1):74-90.

9. Hipp JR, Bauer DJ. Local solutions in the estimation of growth mixture models. Psychol Methods. 2006;11(1):36-53.

10. Inker LA, Eneanya ND, Coresh J, Tighiouart H, Wang D, Sang Y, et al. New Creatinine- and Cystatin C-Based Equations to Estimate GFR without Race. N Engl J Med. 2021;385(19):1737-49.

11. Musso CG, Oreopoulos DG. Aging and physiological changes of the kidneys including changes in glomerular filtration rate. Nephron Physiol. 2011;119 Suppl 1:p1-5.

12. Weinstein JR, Anderson S. The aging kidney: physiological changes. Adv Chronic Kidney Dis. 2010;17(4):302-7.

13. Poggio ED, Rule AD, Tanchanco R, Arrigain S, Butler RS, Srinivas T, et al. Demographic and clinical characteristics associated with glomerular filtration rates in living kidney donors. Kidney Int. 2009;75(10):1079-87.

14. Eriksen BO, Stefansson VTN, Jenssen TG, Mathisen UD, Schei J, Solbu MD, et al. Elevated blood pressure is not associated with accelerated glomerular filtration rate decline in the general non-diabetic middle-aged population. Kidney Int. 2016;90(2):404-10.

15. Proust-Lima C, Joly P, Dartigues J-F, Jacqmin-Gadda H. Joint modelling of multivariate longitudinal outcomes and a time-to-event: a nonlinear latent class approach. Computational Statistics and Data Analysis. 2009;53(4):1142-54.

**Supplementary Table S1.** Cumulative incidences of all-cause mortality for trajectories of eGFR

| **Event** | **Slow eGFR decline** | **Intermediate eGFR decline** | **Fast eGFR decline** | **Increase/stable eGFR** |
| --- | --- | --- | --- | --- |
| **All-cause mortality** | *(N = 8,954)* | *(N = 895)* | *(N = 222)* | *(N = 1,991)* |
| Age 50 | 1.15 (0.71;1.81) | 0.02 (0.007;0.05) | 1.53 (0.49;3.95) | 0.07 (0.03;0.15) |
| Age 60 | 7.48 (4.74;11.8) | 0.47 (0.21;1.05) | 15.0 (6.66;27.7) | 1.00 (0.51;1.89) |
| Age 70 | 32.3 (21.4;47.9) | 6.74 (3.48;12.4) | 68.8 (44.4;87.8) | 9.45 (5.52;15.7) |
| Age 80 | 79.3 (61.5;93.7) | 50.9 (32.5;72.6) | 100 (99.9;100) | 51.4 (34.7;70.9) |

Values are cumulative incidences (%) with 95% confidence bands for the mean of covariates for continuous covariates and the reference category for categorical covariates. Included covariates are age at baseline, sex, Rotterdam Study cohort, body mass index, smoking, alcohol use, cholesterol, triglycerides, hypertension, history of cardiovascular disease, and diabetes. Confidence bands are calculated using a Monte Carlo method.

Abbreviations: eGFR, estimated glomerular filtration rate; N, number.

**Supplementary Table S2.** Cumulative incidences of all-cause mortality for trajectories of eGFR, adjusted for time-varying hypertension and time-varying use of RAAS modifying agents

| **Time-varying hypertension*** | | | | |
| --- | --- | --- | --- | --- |
| **Event** | **Slow eGFR decline** | **Intermediate eGFR decline** | **Fast eGFR decline** | **Increase/stable eGFR** |
| **All-cause mortality** | *(N = 8,954)* | *(N = 895)* | *(N = 222)* | *(N = 1,991)* |
| Age 50 | 1.15 (0.71;1.80) | 0.02 (0.007;0.05) | 1.53 (0.51;3.92) | 0.07 (0.03;0.13) |
| Age 60 | 7.53 (4.70;11.5) | 0.47 (0.20;0.97) | 14.8 (6.94;27.1) | 1.01 (0.52;1.73) |
| Age 70 | 32.5 (21.4;46.2) | 6.60 (3.42;11.9) | 68.2 (46.6;86.3) | 9.48 (5.62;14.9) |
| Age 80 | 79.7 (61.7;92.5) | 50.6 (32.3;70.5) | 99.8 (97.7;100) | 51.4 (35.2;68.8) |
|  |  |  |  |  |
| **Time-varying RAAS modifying agents use **** | | |  |  |
| **Event** | **Slow eGFR decline** | **Intermediate eGFR decline** | **Fast eGFR decline** | **Increase/stable eGFR** |
| **All-cause mortality** | *(N = 8,954)* | *(N = 895)* | *(N = 222)* | *(N = 1,991)* |
| Age 50 | 1.12 (0.73;1.88) | 0.02 (0.006;0.05) | 1.45 (0.48;3.90) | 0.07 (0.03;0.15) |
| Age 60 | 7.70 (4.86;12.0) | 0.48 (0.20;1.00) | 14.4 (6.55;27.3) | 1.01 (0.54;1.90) |
| Age 70 | 33.2 (22.3;48.5) | 6.79 (3.42;12.5) | 68.4 (45.1;86.9) | 9.63 (5.75;15.9) |
| Age 80 | 80.4 (63.5;93.4) | 51.1 (32.5;71.9) | 99.8 (97.3;100) | 52.4 (36.3;70.8) |

Values are cumulative incidences (%) with 95% confidence bands for the mean of covariates for continuous covariates and the reference category for categorical covariates. Confidence bands are calculated using a Monte Carlo method.

*Included covariates are age at baseline, sex, Rotterdam Study cohort, body mass index, smoking, alcohol use, cholesterol, triglycerides, **time-varying hypertension**, history of cardiovascular disease, and diabetes.

**Included covariates are age at baseline, sex, Rotterdam Study cohort, body mass index, smoking, alcohol use, cholesterol, triglycerides, **time-varying RAAS modifying agents use**, history of cardiovascular disease, and diabetes.

Abbreviations: eGFR, estimated glomerular filtration rate; N, number; RAAS, renin-angiotensin-aldosterone system.

**Supplementary Table S3.** All-cause mortality risk for trajectories of eGFR

|  | **Events / total N** | **HR (95% CI)**  **Model 1** | **HR (95% CI)**  **Model 2** |
| --- | --- | --- | --- |
| **Slow decline** | 927 / 2,431 | 1.00 (reference) | 1.00 (reference) |
| **Fast decline** | 106 / 177 | 1.86 (1.52;2.27) | 1.71 (1.40;2.10) |
| **Increase/stable** | 1,079 / 3,084 | 0.93 (0.85;1.01) | 0.94 (0.86;1.03) |

Model 1 is adjusted for age at baseline, sex, and Rotterdam Study cohort

Model 2 is additionally adjusted for body mass index, smoking, alcohol use, cholesterol, triglycerides, hypertension, history of cardiovascular disease, and diabetes

Abbreviations: CI, confidence interval; eGFR, estimated glomerular filtration rate; HR, hazard ratio; N, number.

**Supplementary Figure S1.** Flowchart of the study population

Study population with informed consent

(n = 14,613)

Participants with a eGFR creatinine measurement available

(n = 13,742)

Participants in the Rotterdam Study

(n = 14,926)

Participants without eGFR creatinine measurements in RS or Star-MDC were excluded (n = 871)

Participants without giving informed consent were excluded (n = 313)

Participants with only eGFR creatinine measurements after end of follow-up* in the Rotterdam Study were excluded (n = 23)

Participants with only eGFR creatinine measurements before start of follow-up in the Rotterdam Study were excluded (n = 1,657)

Final study population

(n = 12,062)

(n measurements of eGFR creatinine = 85,922)

*End of follow-up: start of dialysis, receiving kidney transplantation or death

Abbreviations: eGFR, estimated glomerular filtration rate; n number.

**Supplementary Figure S2.** Trajectories of eGFR calculated with the CKD-EPI 2021 equation across age based on serum creatinine levels

**
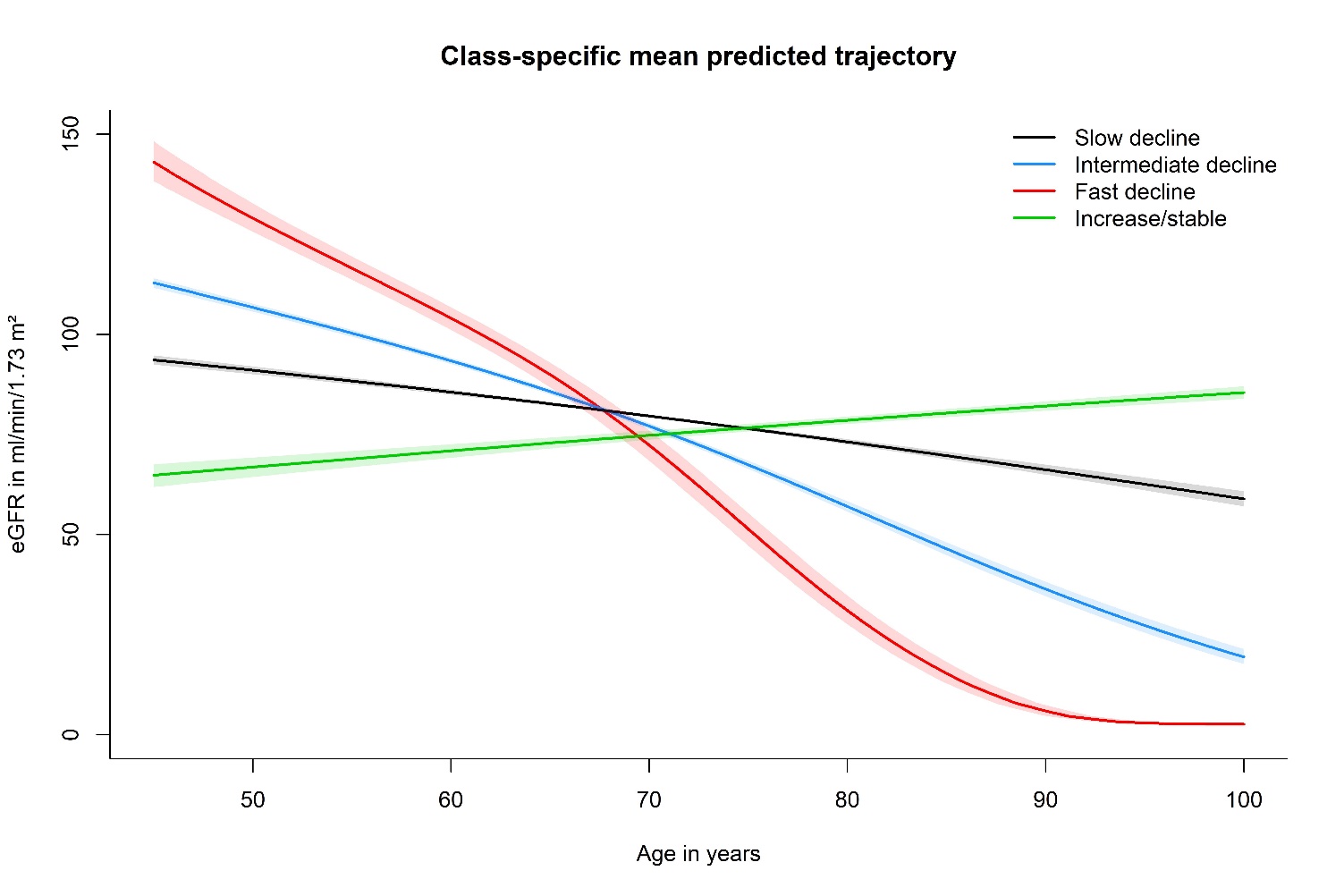
**

Abbreviations: CKD-EPI, Chronic Kidney Disease Epidemiology Collaboration; eGFR, estimated glomerular filtration rate.

**Supplementary Figure S3.** Trajectories of eGFR across age based on serum creatinine levels, taking hypertension as time-varying covariate into account.


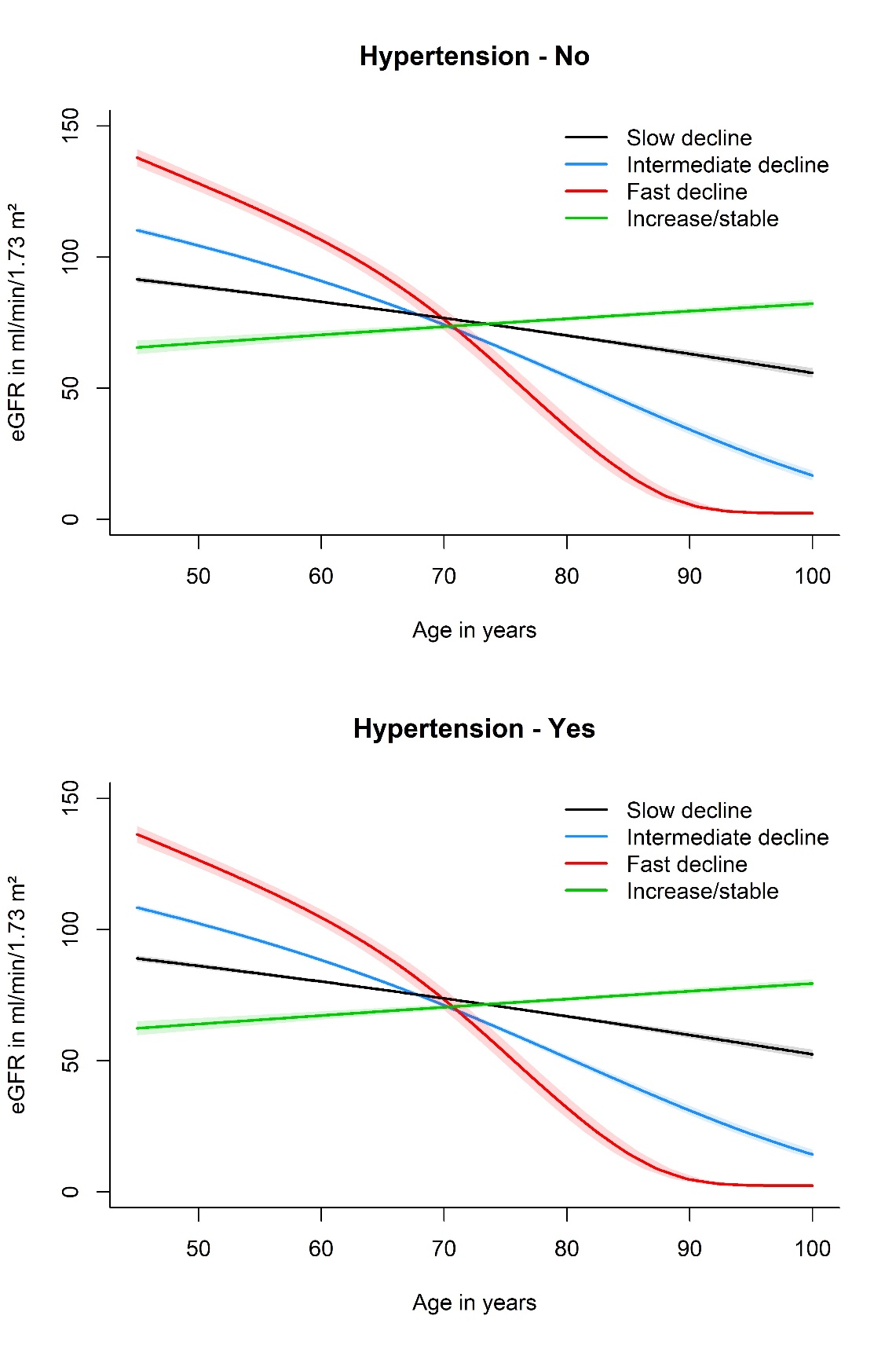


Abbreviations: eGFR, estimated glomerular filtration rate.

**Supplementary Figure S4.** Trajectories of eGFR across age based on serum creatinine levels, taking the use of RAAS modifying agents as time-varying covariate into account.

**
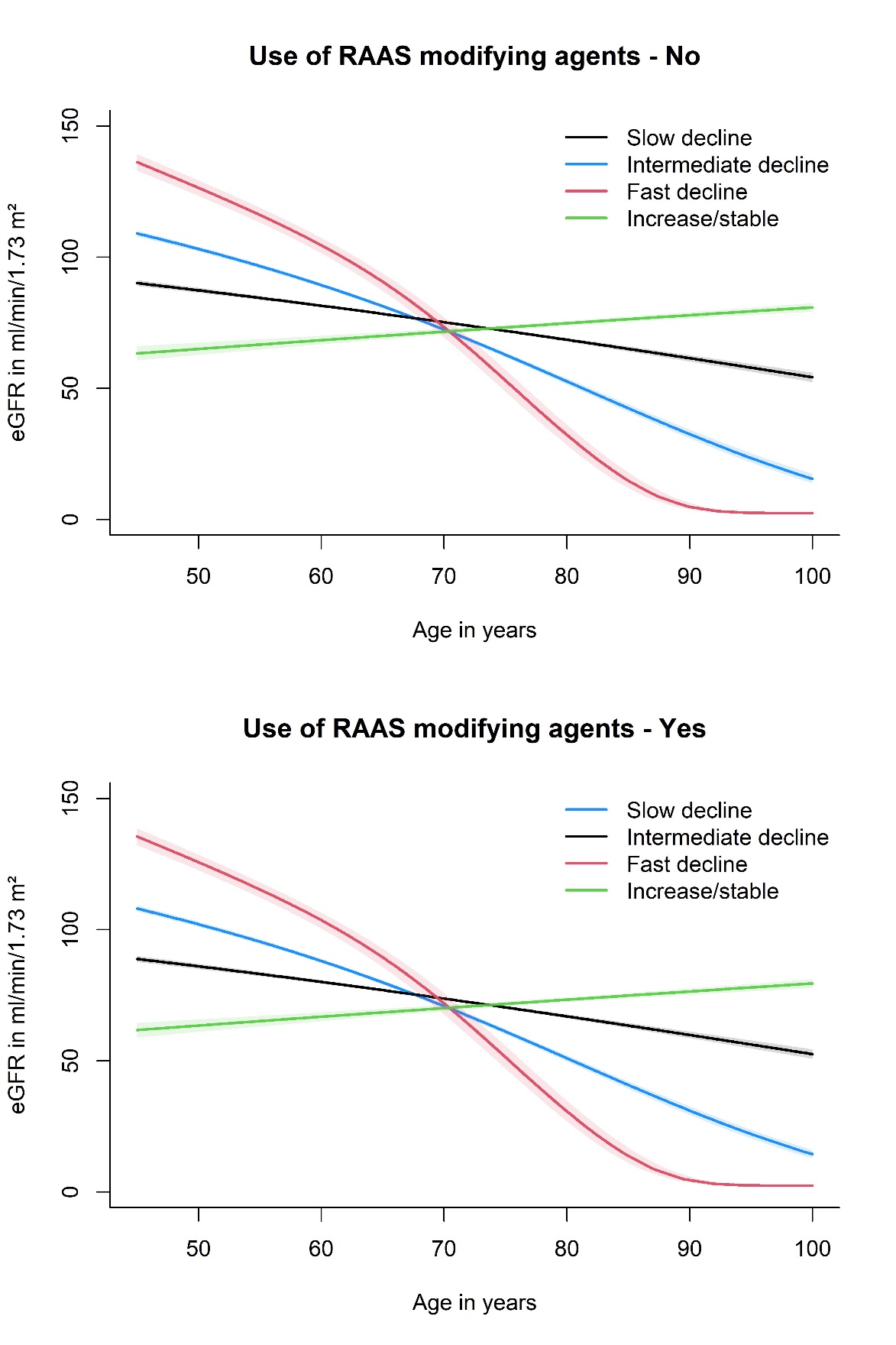
**

Abbreviations: eGFR, estimated glomerular filtration rate; RAAS, renin-angiotensin-aldosterone system.

**Supplementary Figure S5.** Trajectories of eGFR across age based on serum creatinine levels, separately for males and females.

| **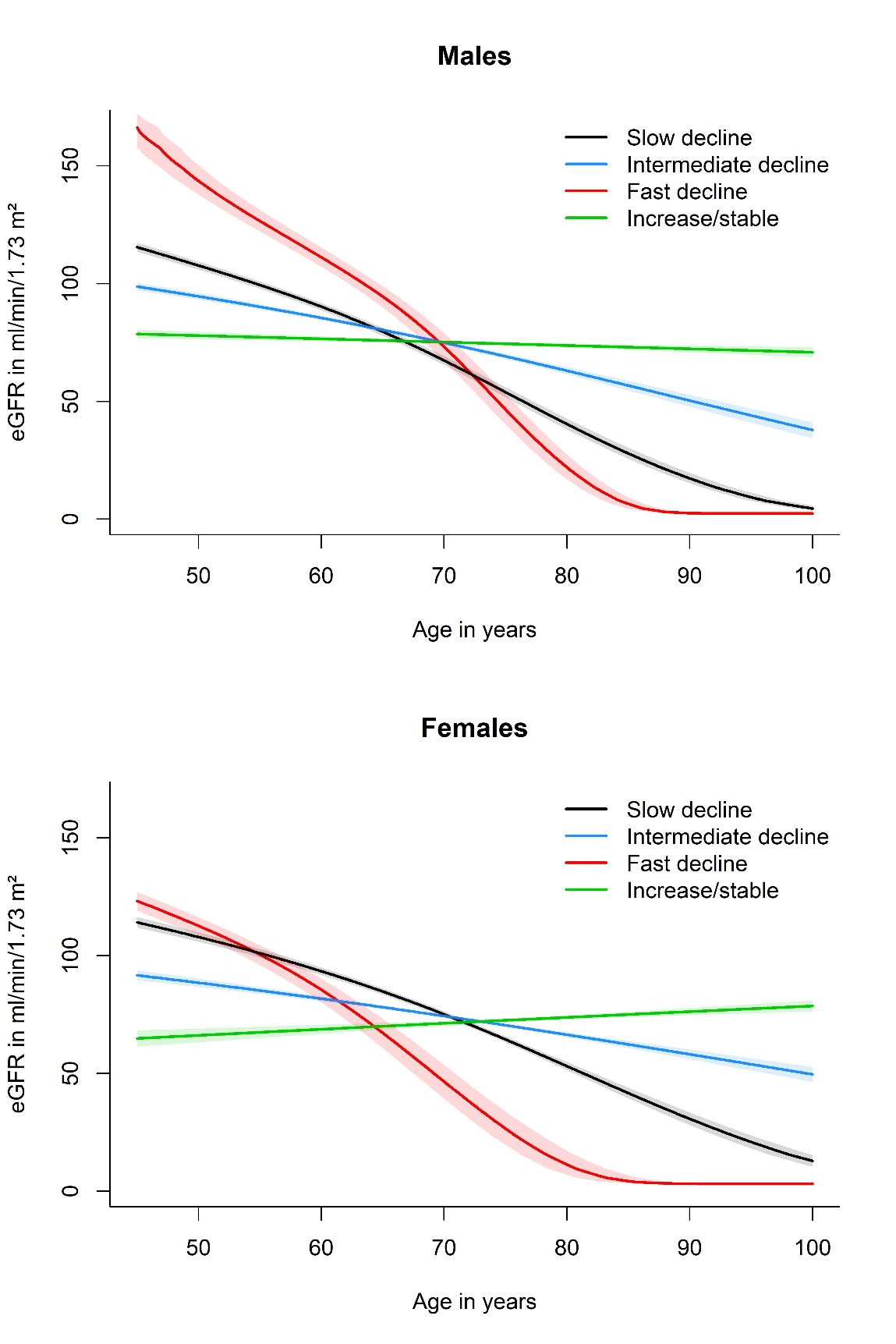** |
| --- |

Abbreviations: eGFR, estimated glomerular filtration rate**.**

**Supplementary Figure S6.** Ten-year trajectories of eGFR across age

**
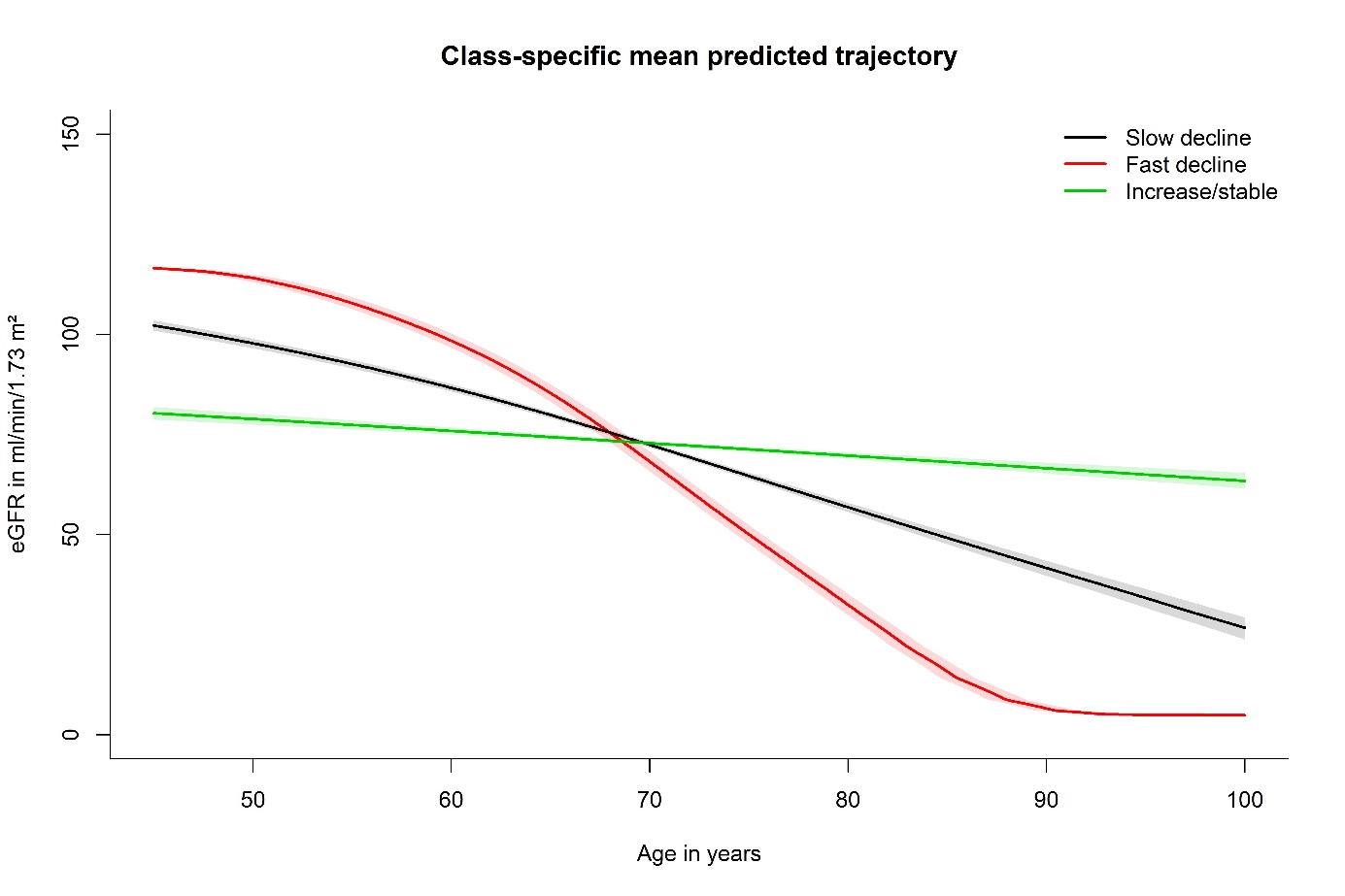
**

Abbreviations: eGFR, estimated glomerular filtration rate.

**Supplemental Figure S7.** Longitudinal evolution of eGFR


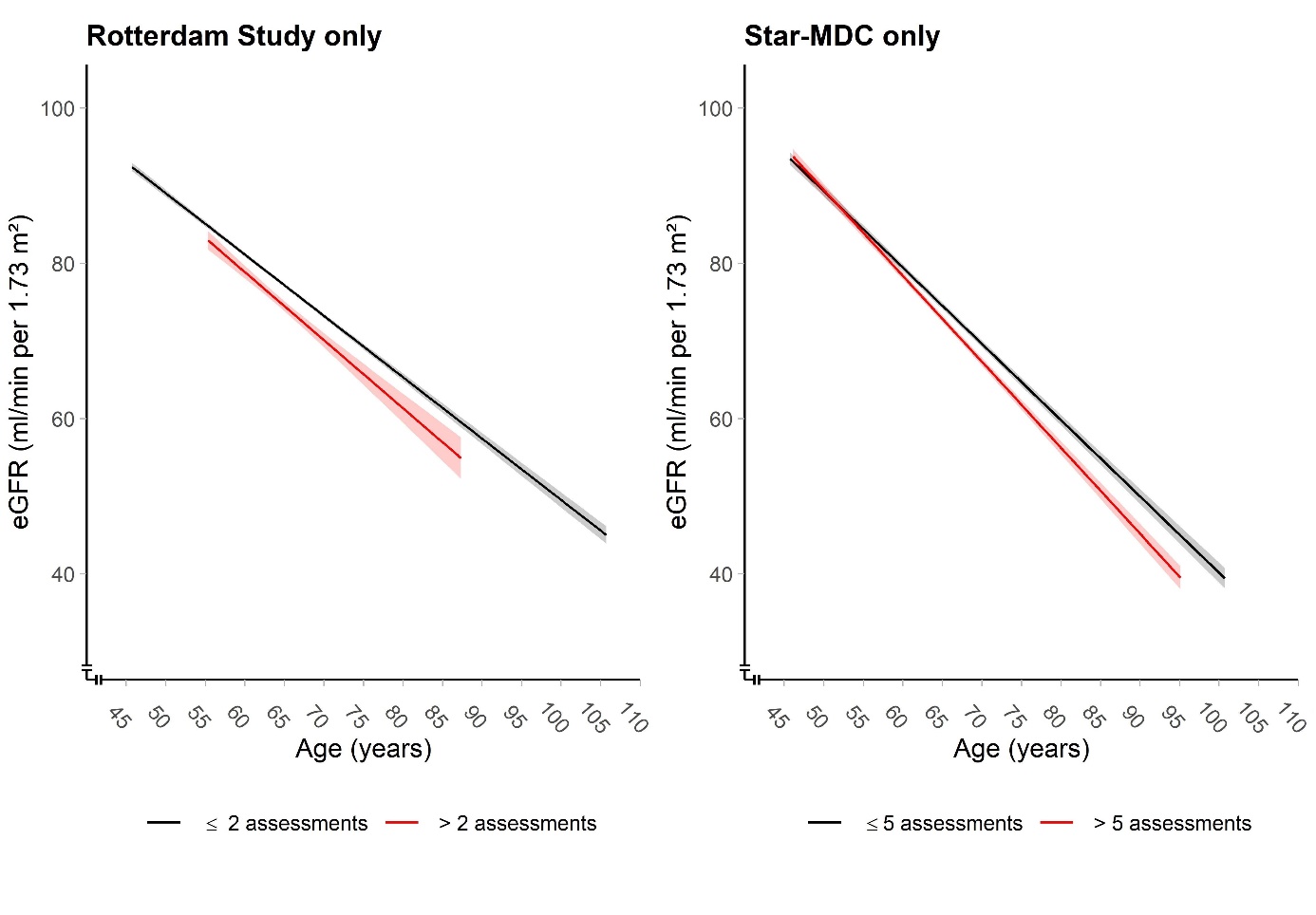


Longitudinal evolution of eGFRcreat with age, shown separately using data from the Rotterdam Study and data from the Star-MDC database. Furthermore, the data is stratified by the number of repeated assessments. Longitudinal eGFR evolutions were similar when using data from the Rotterdam Study and when using data from the Star-MDC database.
